# Trajectories of social participation and risk of cognitive impairment in Chinese older adults: A six-year longitudinal study

**DOI:** 10.1016/j.tjpad.2026.100499

**Published:** 2026-01-30

**Authors:** Kangle Wang, Ruihan Wan, Jiale Peng, Huanghao Zhou, Kaifeng Xu, Hao Liu, Lidian Chen, Zhizhen Liu

**Affiliations:** a College of Rehabilitation Medicine, Fujian University of Traditional Chinese Medicine, Fuzhou, Fujian, China; b Department of Rehabilitation Medicine, Zhongda Hospital Southeast University, Nanjing, Jiangsu, China; c Department of Rehabilitation Medicine, Baiyun District People's Hospital of Guangzhou, Guangzhou, Guangdong, China; d First Clinical Medical College, Henan University of Chinese Medicine, Zhengzhou, Henan, China; e National-Local Joint Engineering Rehabilitation Medicine Technology, Fujian University of Traditional Chinese Medicine, Fuzhou, Fujian, 350122, China; f Fujian Key Laboratory of Rehabilitation Technology, Affiliated Rehabilitation Hospital of Fujian University of Traditional Chinese Medicine, Fuzhou, Fuzhou, 350001, China; g Fujian Key Laboratory of Cognitive Rehabilitation, Affiliated Rehabilitation Hospital of Fujian University of Traditional Chinese Medicine, Fuzhou, Fuzhou, 350001, China

**Keywords:** Older adults, Social participation, Longitudinal study, Cognitive impairment, Group-based trajectory modeling

## Abstract

**Background:**

The growing burden of cognitive decline represents a significant public health concern in aging populations, particularly in China. Social participation is a modifiable factor that may protect against cognitive decline, yet its long-term dynamic association with cognitive impairment remains insufficiently characterized.

**Objectives:**

This study aimed to delineate long-term trajectories of social participation and determine their association with cognitive impairment in Chinese older adults.

**Design:**

**:** Longitudinal cohort study.

**Setting:**

The study utilized data collected in 2013, 2015, and 2018 from the China Health and Retirement Longitudinal Study.

**Participants:**

We included 3074 Chinese adults aged ≥60 years who were free of cognitive impairment in 2013, had complete social participation data in 2013/2015/2018, and completed cognitive assessments in 2018

**Intervention(s):**

Not applicable.

**Measurements:**

Social participation was derived from CHARLS self-reported activity items and frequency and summed into a composite score (range 0–33). Cognitive performance was assessed using episodic memory (immediate and delayed 10-word recall) and mental status (orientation, serial subtraction, and figure drawing), yielding a global score (range 0–31); cognitive impairment was defined as a score <11. Group-based trajectory modeling identified five social participation trajectories. Multivariable logistic regression estimated odds ratios (ORs) for cognitive impairment adjusting for sociodemographic, health, and behavioral covariates.

**Results:**

Five distinct social participation trajectories were identified. In the fully adjusted model, relative to the “stable low” group, those in the “low baseline–increasing” (OR = 0.66, 95% CI: 0.47–0.92), “stable intermediate” (OR = 0.75, 95% CI: 0.58–0.97), and “stable high” (OR = 0.41, 95% CI: 0.22–0.76) groups had markedly reduced chances of cognitive impairment, while no significant link was found for the “moderate decline” group (OR = 0.90, 95% CI: 0.71–1.17).

**Conclusions:**

Maintaining or increasing one’s social activities was linked to a notably lower likelihood of cognitive decline. These results highlight the importance of social involvement patterns as a modifiable factor for fostering cognitive strength. Interventions to maintain or enhance participation are therefore a viable strategy for the primary prevention of cognitive decline in older adults.

## 1 Introduction

With the rapid rise in the global aging population, increasing emphasis has been placed on identifying modifiable determinants that could influence cognitive decline and dementia risk. Currently, around 55 million people are affected by dementia worldwide, with the vast majority residing in developing countries [1]. Among them, China is undergoing one of the most rapid demographic shifts. According to recent estimates, cognitive impairment affects approximately 15.5 % of Chinese older adults, amounting to nearly 40 million individuals and placing a substantial burden on the country’s healthcare system [2]. In response to these challenges, the World Health Organization has advocated for the concept of “active aging,” emphasizing greater engagement in social activities as a potential strategy to mitigate cognitive deterioration [3,4].

Social participation reflects how often and how broadly individuals engage in various social or community-based pursuits, including leisure, volunteerism, interpersonal communication, and engagement with digital platforms [5,6]. The frequency and breadth of these activities may influence cognitive health and protect cognitive function. This protective effect may arise from multiple mechanisms, including enhanced cognitive reserve [7], mental stimulation from diverse interactions [8], maintenance of cerebral blood flow [9], and stronger social support networks that provide emotional and practical assistance [10]. Prior research has highlighted the crucial role of social participation in maintaining cognitive function. For instance, evidence from an 18-year longitudinal study indicates that declining leisure activity participation may serve as an early indicator of dementia [11]. Likewise, a population-based cohort study involving 9936 adults aged 70 years or older found that individuals with higher levels of social engagement were associated with a lower risk of dementia [12]. Moreover, recent studies have reported a nonlinear inverse relationship between the frequency of social interactions and the risk of cognitive decline and depressive symptoms, suggesting that depression may play a mediating role in this association [13]. Nevertheless, most earlier studies have relied on cross-sectional or single-timepoint designs, which cannot capture changes and long-term trajectories of social engagement across the life course. Therefore, longitudinal approaches are needed to characterize these patterns more comprehensively.

To address these limitations, trajectory modeling approaches, particularly group-based trajectory modeling (GBTM), have emerged as useful tools for studying health behaviors [14]. These methods identify distinct longitudinal trajectories within individuals, providing a framework to examine relationship between behavioral dynamics and health outcomes. GBTM and related approaches have been used to examine how long-term social participation relate to health outcomes. For instance, a CHARLS-based study identified subgroups of older adults based on composite measures of social participation and found that individuals following a “high baseline–increasing tendency” trajectory were more likely to maintain functional capacity over time, suggesting potential benefits of sustained social participation [15]. Similarly, analysis using the Chinese longitudinal healthy longevity survey (CLHLS) dataset delineated four distinct trajectories of social participation and reported that psychological well-being, cognitive function, and physical capacity were associated with these patterns [16]. Another investigation using six waves of data from the Korean Longitudinal Study of Aging (KLoSA), applied latent class growth modeling and found that maintaining a greater variety of social activities was associated with a slower rate of cognitive decline [17]. Overall, trajectory-based studies suggest that sustained social participation is associated with more favorable aging outcomes. However, cross-study comparability remains constrained by methodological differences—particularly in how social participation is constructed and scored [15,17], the trajectory modeling approach [15–17], and eligibility criteria for analytic samples [[15], [16], [17], [18]]. These differences may affect trajectory solutions and effect estimates and raise concerns about selection and reverse causation, limiting direct comparisons across studies. Moreover, most studies used continuous cognitive performance scores as outcome measures [17], whereas clinically interpretable endpoints such as incident cognitive impairment have been less frequently examined. Therefore, longitudinal studies are needed to test whether distinct social participation trajectories are associated with incident cognitive impairment.

To address these research gaps, this analysis relies on follow-up data from three survey rounds (2013, 2015, and 2018) conducted under the CHARLS project. A comprehensive social participation index, incorporating both the type of activity and its frequency, was developed and examined. We applied group-based trajectory modeling (GBTM) to identify distinct longitudinal patterns in social participation among Chinese adults aged 60 years and older. Building on the identified trajectory groups, this research investigates their prospective relationship with subsequent cognitive impairment. The analysis accounts for a wide array of covariates, encompassing sociodemographic factors, health-related behaviors, physical function, and depressive symptoms. By doing so, the study seeks to elucidate the longitudinal impact of changing social participation patterns on cognitive health.

## 2 Methods

### 2.1 Participants

This study drew upon three waves of data from the CHARLS, collected in 2013, 2015, and 2018, with the 2013 serving as the baseline. CHARLS is a nationally representative longitudinal survey conducted by the National School of Development at Peking University. It adopts a multistage, stratified sampling strategy to ensure national representativeness. The baseline sample was drawn from 150 counties and 450 urban or rural communities across 28 provinces in China [19], comprising 14,277 respondents who completed face-to-face interviews in 2013. All participants provided written informed consent prior to participation, and the study protocol received ethical clearance from the Institutional Review Board of Peking University (IRB00001052–11,015).

Supplementary Figure S1 illustrates how participants were screened and selected for inclusion in the study. Starting from the initial 14,277 respondents surveyed in 2013, we excluded 10,503 individuals based on the following criteria: being younger than 60 years (*n* = 7592); missing key demographic information such as age, sex, or residence (*n* = 10); lacking baseline data on social participation (*n* = 446) or cognitive function (*n* = 475); having a history of stroke or emotional/psychiatric disorders (*n* = 344); or exhibiting baseline cognitive impairment, as indicated by a baseline cognitive score below 11, based on established thresholds [20] (*n* = 1636). After these exclusions, 3774 participants with complete baseline data remained eligible. We further removed 78 participants due to incomplete social participation data in either 2015 or 2018, and an additional 622 due to missing cognitive assessments in 2018. The final analytic sample comprised 3074 older adults. In addition, to evaluate potential selection bias, we compared baseline characteristics between eligible participants aged ≥60 years who were included in the analytic sample and those who were excluded from the final analysis. The comparison results are presented in Supplementary Table S1.

### 2.2 Social participation assessment

The assessment of social participation was based on participants’ responses to a pair of self-reported questions. The first question asked: “Did you take part in any of the following activities during the past month?” The first question covered 12 categories of activities: (1) meeting with friends; (2) playing mahjong, chess, cards, or joining community clubs; (3) offering assistance to non-cohabiting family, friends, or neighbors without financial compensation; (4) being involved in sports, social, or other clubs; (5) engaging in community-related groups; (6) participating in voluntary or charitable services; (7) caring for a sick or disabled adult who does not reside with the respondent and without pay; (8) attending educational or training sessions; (9) investing in the stock market; (10) using the internet; (11) engaging in other listed activities; and (12) none of the above.

For individuals who reported engagement in any of the initial 11 activity types, a follow-up question assessed how frequently they participated. Response options were classified into three levels: (1) almost daily, (2) almost weekly, and (3) irregularly, corresponding to scores of 3, 2, and 1, respectively. If the respondent chose “none of the above,” a score of 0 was assigned. A total social participation index was derived by aggregating the frequency values reported for each activity, resulting in a continuous measure ranging from 0 to 33. This composite index has been used in prior CHARLS-based research, with reported internal consistency across waves (Cronbach’s α = 0.591–0.609) [21].

### 2.3 Cognitive function assessment

Cognitive function was assessed across two domains: episodic memory and mental status. For memory evaluation, participants completed an immediate and delayed recall task using a randomly ordered 10-word list. Scores ranged from 0 to 20 based on the total number of correct responses. Mental status was measured using items adapted from the Telephone Interview for Cognitive Status (TICS), including orientation (e.g., date and season), serial subtraction of 7 from 100, and a figure-copying task. Each correct response received 1 point, yielding a total score ranging from 0 to 11. Global cognitive performance was represented by summing the two domain scores (range: 0–31), with higher values indicating better function [22]. Individuals with total scores below 11 were classified as cognitively impaired [23,24].

### 2.4 Covariates

In the regression analysis, we adjusted for a prespecified set of baseline (2013) covariates, including sociodemographic attributes, physical and mental health indicators, and behavioral factors. The demographic characteristics comprised age, sex (male or female), marital status (married or others), educational level (illiterate, primary school, junior high, senior high or above), and residence (city or rural). Health-related variables encompassed body mass index (BMI, grouped as underweight, normal, overweight, or obese), functional status as measured by activities of daily living (ADL) and instrumental activities of daily living (IADL), chronic disease history (yes or no), depressive symptoms (evaluated using the CESD-10 scale), and self-rated health. Behavioral factors included current smoking and alcohol use, both coded as binary variables (yes/no). Missing covariate data were addressed via multiple imputation to reduce bias.

### 2.5 Statistical analysis

GBTM is a statistical approach designed to uncover latent subpopulations within longitudinal data by identifying individuals with similar developmental patterns over time [14]. In the present analysis, GBTM was applied to characterize social participation trajectories across three survey waves (2013, 2015, and 2018). A zero-inflated Poisson distribution was used to model the social participation scores. Trajectories were estimated and visualized using the traj and trajplot commands in Stata. Models incorporating one to five distinct groups were assessed. Each specification included intercept, linear, and quadratic terms for time. Model selection followed three main principles: (1) preference for models with lower Bayesian Information Criterion (BIC) values; (2) average posterior probabilities (AvePP) exceeding 0.7 in each class; and (3) ensuring that no group accounted for less than 5 % of the total sample [25].

Baseline differences between individuals with and without cognitive impairment were evaluated using descriptive statistics. Continuous variables conforming to a normal distribution were described using means and standard deviations, with comparisons conducted through independent-samples *t*-tests. For non-normally distributed data, medians and interquartile ranges were reported, and group differences were assessed using nonparametric rank-sum tests. Frequencies and relative proportions were calculated for categorical variables, and intergroup comparisons were performed using the chi-square test. Multinomial logistic regression was used to examine baseline determinants of trajectory-group membership. The association between social participation trajectory patterns and cognitive impairment was examined using binary logistic regression. Model 1 included only the trajectory variable (unadjusted). Model 2 additionally adjusted for sociodemographic factors (age, sex, residence, education, and marital status). Model 3 further incorporated health behaviors (smoking and drinking status), and Model 4 added BMI, functional ability (ADL and IADL), depressive symptoms, chronic conditions, and self-rated health.

Two sensitivity analyses were performed: (1) baseline cognitive score (2013) was additionally included in the fully adjusted model (Model 4); and (2) the fully adjusted analysis was repeated among participants with complete covariate information (complete-case analysis; *n* = 1326). Statistical procedures were carried out using IBM SPSS (version 27.0) and Stata (version 18.0), with a two-tailed p-value < 0.05 considered indicative of significance.

## 3 Results

### 3.1 Social participation trajectories

A total of 3074 individuals aged 60 or older, who showed no signs of cognitive impairment at baseline, were ultimately included in the final analytic sample. Using the GBTM approach, this study fitted models with varying numbers of trajectory groups and polynomial specifications. As shown in Supplementary Table S2, increasing the number of trajectory groups was associated with progressively lower BIC values. After evaluating BIC, AIC, entropy values, and the AvePP across groups, the five-group trajectory model (0 1 1 0 0) was identified as providing the best overall fit.

**Fig. 1:**
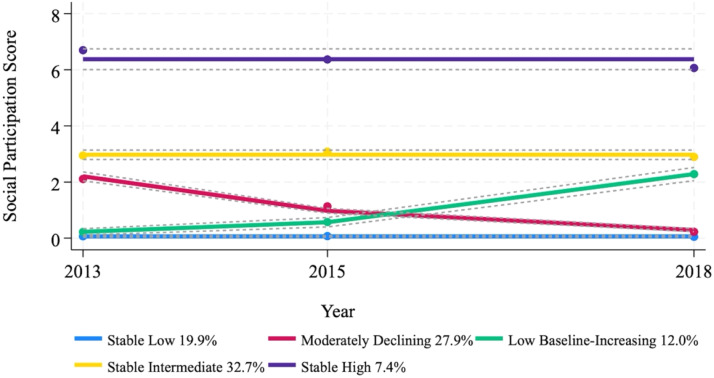
**Estimated social participation trajectories over a six-year follow-up period**. Note: Solid lines depict model-estimated trajectories; dots represent observed group means at each wave; dashed lines indicate the 95 % confidence intervals for the estimated values. Percentages in the legend represent model-estimated group proportions (TotProb). Group sizes based on maximum posterior assignment are reported in the main text.

Fig. 1 illustrates the five longitudinal social participation trajectories and their relative sizes: stable low (*n* = 628, 20.4 %), moderately declining (*n* = 879, 28.6 %), low baseline–increasing (*n* = 349, 11.4 %), stable intermediate (*n* = 1021, 33.2 %), and stable high (*n* = 197, 6.4 %). The stable low group maintained social participation scores close to zero throughout the follow-up period, indicating minimal or no engagement in social activities. The moderately declining group started at a moderate level (approximately 2.3 points) but exhibited a linear decline over time. The low baseline-increasing group began with a low score but increased gradually, reaching approximately 2.5 points by 2018. The stable intermediate group consistently maintained moderate participation levels (approximately 3 points) across the three time points. In contrast, the stable high group exhibited persistently high social participation, with scores remaining above 6.5 points throughout the follow-up.

### 3.2 Baseline characteristics

Over the six-year follow-up, 759 individuals were newly diagnosed with cognitive impairment in 2018, corresponding to an incidence rate of 24.7 %. Table 1 presents baseline characteristics of individuals who later exhibited cognitive impairment (CI group) compared with those who maintained normal cognitive function (NCI group). Individuals who developed cognitive impairment by 2018 were more likely to belong to the stable low and moderately declining social participation trajectories than those who remained cognitively normal. These individuals were more often female, older, living in rural areas, unmarried or widowed, less educated, functionally impaired, more depressed, and reported poorer self-rated health.

**Table 1:** Baseline characteristics of participants by cognitive impairment status in 2018.

Characteristics	CI	NCI	P value
Age	65(62,70)	65(62,68)	<0.001
Sex			<0.001
Male	356(46.9 %)	1417(61.2 %)	
Female	403(53.1 %)	898(38.8 %)	
Residence			
Rural	734(96.7 %)	2001(86.4 %)	
City	25(3.3 %)	314(13.6 %)	
BMI			<0.001
Lean	62(8.3 %)	99(4.4 %)	
Normal	491(65.5 %)	1431(62.9 %)	
Overweight	168(22.4 %)	632(27.8 %)	
Obese	29(3.9 %)	113(5.0 %)	
Education			<0.001
Illiterate	295(38.9 %)	165(7.1 %)	
Primary school	413(57.6 %)	1357(58.6 %)	
Junior high school	40(5.3 %)	505(21.8 %)	
Senior high school and above	11(1.4 %)	288(12.4 %)	
Marital status			<0.001
Married	626(82.5 %)	2046(88.4 %)	
Other	133(17.5 %)	269(11.6 %)	
ADL	6(5,6)	6(6,6)	0.146
IADL	6(5,6)	6(6,6)	<0.001
CESD-10	7(4,12)	6(3,10)	<0.001
Smoking			0.872
Yes	157(20.9 %)	470(20.7 %)	
No	593(79.1 %)	1805(79.3 %)	
Drink			0.011
Yes	266(35.0 %)	931(40.2 %)	
No	493(65.0 %)	1384(59.8 %)	
Self-rated health status	4(3,4)	3(3,4)	0.001
Chronic disease			0.893
Yes	171(22.5 %)	527(22.8 %)	
No	588(77.5 %)	1788(77.2 %)	
Cognitive	14(12,16)	17(15,20)	<0.001
Social participation trajectories			<0.001
Stable low	203(32.3 %)	425(67.7 %)	
Moderately declining	242(27.5 %)	637(72.5 %)	
Low baseline–increasing	82(23.5 %)	267(76.5 %)	
Stable intermediate	217(21.3 %)	804(78.7 %)	
Stable high	15(7.6 %)	182(92.4 %)	

Note: data are expressed as median (IQR) for non-normally distributed continuous variables and as counts (percentages) for categorical variables. For social participation trajectories, percentages are calculated within each trajectory group (row percentages: CI/[CI+NCI]). P-values were obtained via Wilcoxon rank-sum test for continuous variables and chi-square test for categorical data. Cognitive status was determined in the 2018 follow-up. Abbreviations: CI = cognitively impaired; NCI = non-cognitively impaired; BMI = Body Mass Index; ADL = activities of daily living; IADL = instrumental activities of daily living; CESD-10 = 10-item Center for Epidemiologic Studies Depression Scale.

### 3.3 Baseline predictors of social participation trajectory membership

To investigate the determinants of social participation patterns, a multinomial logistic regression model was applied (Table 2), with the stable intermediate group serving as the reference category because it exhibited consistently moderate engagement across waves. Findings indicated that, relative to those following a stable intermediate pattern, individuals who were male, resided in rural areas, experienced limitations in ADL/IADL, showed more severe depressive symptoms, and attained lower levels of education were more likely to belong to the stable low or moderately declining trajectories. In contrast, individuals with higher educational attainment, urban residence, fewer depressive symptoms, and moderate alcohol consumption were more likely to be classified into the stable high trajectory group. Additionally, marital status played a role—those who were unmarried or widowed were less likely to be classified into the stable low or low baseline–increasing groups.

**Table 2:** Multinomial logistic regression analysis of factors associated with social participation trajectories (ref: stable intermediate group).

Characteristics	Stable low		Moderately declining		Low baseline–increasing			Stable high	
	OR (95 % CI)	P value	OR (95 % CI)	P value	OR (95 % CI)	P value	OR (95 % CI)		P value
Age	1.01(0.99,1.04)	0.24	1.02(1.00,1.04)	0.088	1.00(0.97,1.03)	0.980	0.98(0.95,1.01)		0.230
Male (ref: female)	1.54(1.19,1.99)	<0.001	1.36(1.08,1.71)	0.008	0.97(0.73,1.34)	0.929	0.52(0.35,0.77)		0.001
Rural (ref: city)	2.80(1.81,4.32)	<0.001	1.78(1.29,2.45)	<0.001	1.39(0.90,2.14)	0.141	0.49(0.34,0.71)		<0.001
BMI (ref: obese)									
Lean	2.21(1.10,4.45)	0.026	1.43(0.80,2.57)	0.228	2.38(1.02,5.62)	0.044	0.73(0.23,2.28)		0.582
Normal	1.84(1.08,3.16)	0.026	1.25(0.82,1.91)	0.309	2.10(1.07,4.12)	0.030	1.04(0.54,2.04)		0.900
Overweight	1.35(0.77,2.36)	0.293	0.96(0.61,1.49)	0.838	1.37(0.68,2.76)	0.377	0.96(0.48,1.92)		0.914
Education (ref: senior high school and above)									
Illiterate	2.71(1.68,4.39)	<0.001	1.89(1.25,2.88)	0.003	1.91(1.06,3.42)	0.031	0.14(0.06,0.32)		<0.001
Primary school	1.91(1.27,2.89)	0.002	1.68(1.19,2.37)	0.003	1.83(1.11,3.01)	0.017	0.32(0.21,0.49)		<0.001
Junior high school	1.22(0.77,1.94)	0.400	1.19(0.81,1.74)	0.382	0.93(0.53,1.66)	0.817	0.56(0.35,0.87)		0.011
Marital status (ref: married)									
Other	0.54(0.38,0.76)	<0.001	0.99(0.75,1.29)	0.918	0.61(0.41,0.92)	0.017	0.94(0.57,1.56)		0.821
ADL	1.21(1.07,1.37)	0.002	1.00(0.91,1.11)	0.943	1.00(0.88,1.14)	0.992	0.86(0.72,1.02)		0.073
IADL	0.83(0.73,0.94)	0.003	1.00(0.88,1.12)	0.932	0.93(0.80,1.09)	0.364	1.10(0.85,1.43)		0.475
CESD-10	1.03(1.01,1.05)	0.005	1.01(0.99,1.03)	0.217	1.02(0.99,1.04)	0.221	0.94(0.90,0.97)		<0.001
Smoke (ref: no)	0.82(0.63,1.08)	0.161	0.94(0.74,1.20)	0.621	0.98(0.70,1.36)	0.888	0.72(0.44,1.16)		0.177
Drink (ref: no)	0.83(0.66,1.05)	0.119	0.89(0.72,1.10)	0.279	0.82(0.61,1.09)	0.169	1.59(1.10,2.29)		0.013
Self-rated health status	0.92(0.82,1.03)	0.124	0.99(0.89,1.09)	0.816	1.01(0.88,1.15)	0.933	0.95(0.80,1.14)		0.582
No chronic disease	1.03(0.80,1.32)	0.844	0.92(0.73,1.16)	0.473	1.02(0.75,1.39)	0.907	0.75(0.49,1.13)		0.170

Note: multinomial logistic regression identified factors related to trajectory group membership, using the Stable Intermediate group as the reference. Covariates included age, sex, residence, education, marital status, BMI, ADL, IADL, depression symptoms, tobacco and alcohol use, self-perceived health, and chronic disease presence. OR = odds ratio; CI = confidence interval.

### 3.4 Association between social participation trajectories and cognitive impairment

**Fig. 2:**
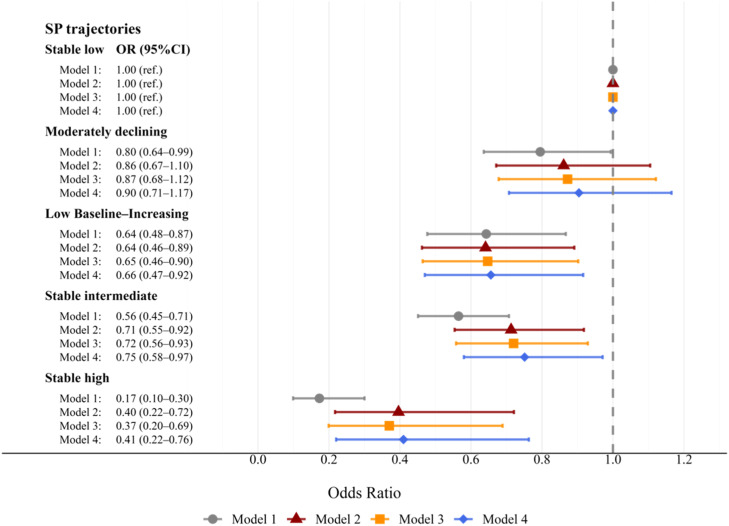
**Forest plot illustrating the association between social participation trajectories and cognitive impairment in 2018**. Note: Model 1 is unadjusted; Model 2 controls for age, sex, residential location (urban vs. rural), marital status, and education; Model 3 additionally includes smoking and alcohol use; Model 4 further adjusts for BMI, ADL, IADL, depressive symptoms, self-rated health, and chronic illness status. OR = odds ratio; CI = confidence interval.

Binary logistic regression models with stepwise covariate adjustments were employed to explore associations between social participation trajectories and cognitive impairment, with the stable low group serving as the reference (Table 3). Fig. 2 summarizes how these ORs change across successive adjustment models. In the unadjusted model, all non-reference trajectory groups were associated with a notably lower odds of cognitive impairment compared to the stable low group (*P* < 0.05). Among them, the stable high group exhibited the strongest protective association (OR = 0.17, 95 % CI: 0.10–0.30, *P* < 0.001). In the fully adjusted model (Model 4), three trajectory groups—low baseline–increasing (OR = 0.66, 95 % CI: 0.47–0.92, *P* = 0.013), stable intermediate (OR = 0.75, 95 %CI: 0.58–0.97, *P* = 0.029), and stable high (OR = 0.41, 95 %CI: 0.22–0.76, *P* = 0.005)—continued to show a statistically significant inverse relationship with cognitive impairment. In contrast, the moderately declining group was not significantly associated with cognitive impairment in Model 4 (*P* = 0.436).

**Table 3:** Logistic regression assessing the relationship between social participation patterns and cognitive impairment risk.

Characteristics		Model 1		Model 2		Model 3		Model 4	
		OR (95 % CI)	P value	OR (95 % CI)	P value	OR (95 % CI)	P value	OR (95 % CI)	P value
Social participation trajectories (ref: stable low group)									
Moderately declining		0.80(0.64,0.99)	0.044	0.86(0.67,1.11)	0.24	0.87(0.68,1.12)	0.285	0.90(0.71,1.17)	0.436
Low baseline–increasing		0.64(0.48,0.87)	0.004	0.64(0.46,0.89)	0.008	0.65(0.46,0.90)	0.01	0.66(0.47,0.92)	0.013
Stable intermediate		0.57(0.45,0.71)	<0.001	0.71(0.55,0.92)	0.009	0.72(0.56,0.93)	0.011	0.75(0.58,0.97)	0.029
Stable high		0.17(0.10,0.30)	<0.001	0.40(0.22,0.72)	0.002	0.37(0.20,0.69)	0.002	0.41(0.22,0.76)	0.005
Age				1.06(1.04,1.08)	<0.001	1.06(1.04,1.08)	<0.001	1.05(1.03,1.07)	<0.001
Female (ref: male)				1.09(0.90,1.33)	0.389	1.30(1.04,1.64)	0.024	1.24(0.98,1.56)	0.073
City (ref: rural)				0.40(0.26,0.64)	<0.001	0.40(0.25,0.63)	<0.001	0.42(0.27,0.67)	<0.001
Married (ref: other)				0.74(0.57,0.96)	0.026	0.76(0.58,0.99)	0.04	0.80(0.61,1.04)	0.099
Education (ref: illiterate)									
Primary school				0.17(0.14,0.22)	<0.001	0.17(0.14,0.22)	<0.001	0.18(0.14,0.23)	<0.001
Junior high school				0.05(0.04,0.08)	<0.001	0.05(0.03,0.07)	<0.001	0.05(0.04,0.08)	<0.001
Senior high school and above				0.03(0.01,0.05)	<0.001	0.02(0.01,0.05)	<0.001	0.03(0.01,0.05)	<0.001
Smoke (ref: no)						1.37(1.07,1.75)	0.011	1.29(1.01,1.65)	0.043
Drink (ref: no)						0.83(0.67,1.03)	0.094	0.83(0.67,1.03)	0.097
BMI (ref: lean)									
Normal								0.58(0.39,0.86)	0.007
Overweight								0.54(0.35,0.83)	<0.001
Obese								0.45(0.25,0.83)	0.01
ADL								1.05(0.95,1.17)	0.312
IADL								0.86(0.77,0.96)	0.005
CESD-10								1.03(1.01,1.05)	0.005
Self-rated health status								1.00(0.90,1.11)	0.972
With chronic disease (ref: no)								0.92(0.73,1.17)	0.501

Note: logistic models estimated the association between different social participation trajectories and cognitive impairment risk, using the stable low group as reference. Model 1 included no covariates; Model 2 adjusted for sociodemographic factors; Model 3 incorporated health behaviors; Model 4 further included physical health and psychological well-being indicators. OR = odds ratio; CI = confidence interval.

When the stable high group was used as the reference (Supplementary Table S3), the stable low trajectory showed the highest odds of experiencing cognitive decline (OR = 2.39, 95 % CI: 1.28–4.45, *P* = 0.006), followed by the moderately declining group (OR = 2.17, 95 % CI: 1.18–4.01, *P* = 0.013). In contrast, the associations for the low baseline–increasing (*P* = 0.184) and stable intermediate (*P* = 0.054) groups did not reach statistical significance. Moreover, multiple covariates such as age, place of residence, smoking behavior, BMI, education level, IADL ability, and depressive symptoms were significantly linked to cognitive impairment risk in 2018.

### 3.5 Sensitivity analysis

As a sensitivity analysis, baseline cognitive score (2013) was additionally included in the fully adjusted model (Supplementary Table S4). The low baseline–increasing trajectory remained significantly associated with lower odds of cognitive impairment compared with the stable low group (OR = 0.68, 95 % CI 0.49–0.96; *P* = 0.030). The other trajectory groups were not significantly associated with cognitive impairment in this model. After excluding observations with missing covariate data, a subsample of 1326 participants was retained for sensitivity analysis. The trajectory modeling based on this reduced sample yielded a similar five-group classification (supplementary Table S5 and Figure S2). Following covariate adjustment, both the stable intermediate and stable high groups continued to exhibit significant protective associations with cognitive impairment relative to the stable low group, whereas the protective effect observed for the low baseline–increasing group was no longer statistically significant (supplementary Table S6)

## 4 Discussion

Drawing on nationally representative CHARLS data, the analysis utilized GBTM to identify five trajectories of social participation among older Chinese adults, including a “low baseline–increasing” pattern. After excluding participants with baseline cognitive impairment and comprehensively adjusting for demographic, behavioral, and health-related covariates, we observed significant associations between social participation patterns and subsequent cognitive impairment. Compared with the “stable low” group, participants in the “low baseline–increasing,” “stable intermediate,” and particularly the “stable high” trajectories showed lower odds of cognitive impairment, with the “stable high” group showing the most pronounced protective effect. In contrast, although the “moderately declining” group began with higher levels of engagement, its downward trend may have attenuated the associated cognitive benefits, resulting in higher odds of cognitive impairment than the stable high group. These results emphasize the preventive potential of sustained or enhanced social participation in preserving cognitive health. In addition, factors such as educational attainment, urban or rural residence, functional status, and depressive symptoms emerged as significant predictors of cognitive impairment.

By identifying five longitudinal trajectories of social engagement among older adults in China, this study provides a more fine-grained characterization of heterogeneity in engagement patterns than some prior trajectory studies. Compared with a nationally representative Korean sample (Kim et al., 2022) [17], we observed a wider range of trajectory shapes in the CHARLS cohort, including a “low baseline–increasing” subgroup that has been less frequently reported in earlier work. Such differences may partly reflect variation in measurement (e.g., the breadth of activity indicators and the use of a frequency-based composite score), the number of waves and follow-up duration, and cohort composition. More broadly, sociocultural and structural contexts in China and South Korea may also shape opportunities and norms for later-life engagement [26]. Compared with the two trajectories identified by Xu et al. (2022) [15] using CHARLS data, our study yielded a more differentiated five-class solution. This discrepancy likely reflects differences in study aims and analytic design (e.g., cohort definition, follow-up structure, and eligibility criteria), which can materially influence class enumeration and the resulting number and shapes of trajectories. Furthermore, these findings align with studies utilizing data from the CLHLS, including studies by Ye et al. (2020) and Zhang et al. (2023), which have reported the existence of three to four distinct trajectories [16,18]. This consistency reinforces the conclusion that substantial variation exists in how Chinese older adults engage in social activities. Collectively, the elaborated trajectory framework established in this work strengthens future research aimed at uncovering how long-term social activity patterns relate to cognitive functioning.

Results showed that those with lower education, male sex, rural background, physical impairments, and pronounced depressive symptoms were more likely to be classified into the “stable low” or “moderately declining” participation groups. This observation aligns with prior investigations into how elderly individuals typically engage in social activities [4,18,27]. Individuals of advanced age with limited education typically experience a range of disadvantages, including reduced access to social resources, a lack of cognitive reserves, and diminished self-efficacy. These factors collectively create barriers that limit both their motivation and capacity to participate socially [28,29]. The association between sex and social participation was also evident, and findings from prior studies have been mixed across settings [18,30,31]. In our analysis, men were more likely to belong to the stable low and moderately declining trajectories and less likely to be classified into the stable high group. This pattern is consistent with CLHLS latent class evidence indicating that men were less likely to be in the “Moderate activity” class [30]. A plausible explanation is that men’s social networks are more closely tied to employment; therefore, the transition into retirement may lead to a sharper loss of work-based ties and a greater decline in participation, as observed in KLoSA [32]. In addition, aging individuals in rural regions face persistent barriers that merit urgent attention. Such individuals encounter a myriad of structural constraints that significantly impact their ability to engage socially. Inadequate infrastructure, lack of organized community services, poor transportation, and limited social networks all create substantial obstacles to social participation [33]. These structural shortcomings, combined with generally lower education levels and constrained access to healthcare, may compound the vulnerability of rural-dwelling seniors to both isolation and deteriorating cognitive functioning [34,35]. To mitigate such disparities, tailored interventions are needed to improve social connectedness and quality of life. Limitations in daily functioning, particularly IADL impairments, clearly limit mobility and participation opportunities in social contexts [36]. Concurrently, elevated depressive symptoms not only undermine social motivation and engagement [37] but also serve as a well-established predictor of cognitive impairment. Collectively, these findings suggest that social participation trajectories are shaped not only by individual factors but also by the interplay of resource accessibility, health status, and broader structural and environmental factors. Identifying these high-risk groups with insufficient social participation is essential for designing and implementing targeted intervention strategies.

After adjustment for a broad range of demographic characteristics, lifestyle behaviors, and health conditions, participants in the “low baseline–increasing,” “stable intermediate,” and “stable high” social participation trajectories exhibited significantly lower odds of cognitive impairment compared with those in the “stable low” group, with the largest magnitude of association observed for the “stable high” trajectory. In contrast, the “moderately declining” trajectory was not significantly different from the “stable low” group in the fully adjusted model. The “stable low” group had the highest cognitive impairment prevalence (32.3 %), indicating that prolonged social disengagement may be associated with poorer cognitive outcomes. This observation is partly in line with Kim et al.’s findings [17] which indicated that participants with consistently high or moderate levels of social activity had slower declines in cognitive performance than those whose engagement declined from a low baseline. Consistently, evidence from Western populations also supports a protective role of sustained social connectedness. For example, in the UK Whitehall II cohort, more frequent social contact in late midlife was associated with a lower subsequent risk of dementia; although dementia differs from cognitive impairment, the finding is consistent with a potential cognitive-reserve pathway [38]. Notably, the “low baseline–increasing” trajectory was associated with lower odds of cognitive impairment compared with the “stable low” group. In a sensitivity analysis additionally adjusting for baseline cognitive performance, the association for the “low baseline–increasing” trajectory remained in the same direction and of comparable magnitude, suggesting that the finding is not solely explained by baseline cognitive differences. While causality cannot be inferred, this pattern may reflect a potentially modifiable profile, either through benefits of increased engagement itself or through correlated improvements in health, functioning, or social resources. One plausible hypothesis is that the increase reflects later-life role transitions or re-socialization processes, such as retirement-related restructuring of daily routines, rebuilding social networks after widowhood, or grandparenting/caregiving that re-embeds older adults into community interactions [32]. Another hypothesis is that the trajectory captures improved opportunity structures, including expanded access to community programs or local policy initiatives that facilitate participation (e.g., organized activities or services), particularly for those initially disengaged [35,37]. Although we could not directly test these triggers in CHARLS, future studies incorporating time-stamped life events and community-level measures could evaluate these competing explanations.

Theoretically, several mechanisms could elucidate the association between social participation and cognitive health. First, the “use it or lose it” hypothesis posits that ongoing cognitive function maintenance necessitates persistent stimulation; social participation may engage higher-order processes such as language, memory, and executive functions [[39], [40], [41]]. Second, engagement in social activities may bolster cognitive reserve by improving neural efficiency and brain plasticity, thereby delaying neurodegeneration [42,43]. Third, social participation promotes physical activity and emotional wellness, potentially leading to improved cerebral blood circulation and decreased chronic inflammation—both key risk factors for dementia [8,13,44]. Finally, social participation promotes interpersonal connections and broader access to support networks, which may help relieve psychological burden and reduce neurobiological alterations associated with stress that contribute to cognitive deterioration [9,13]. Depressive symptoms were also an independent predictor of cognitive impairment, suggesting that emotional well-being may represent a plausible pathway linking social participation to cognitive health. Future work could evaluate this hypothesis using longitudinal mediation analyses and methods that account for time-varying confounding.

Beyond these empirical findings, this study offers both methodological and practical contributions. Using nationally representative CHARLS data and GBTM, we identified five distinct longitudinal trajectories of social participation, including a “low baseline–increasing” subgroup that has been less frequently reported and may reflect a potentially modifiable engagement profile. By linking these trajectories to subsequent cognitive impairment, our findings support the utility of trajectory-based characterization for risk stratification and suggest that changes in engagement over time may be relevant for cognitive outcomes. From a public-health perspective, trajectory-informed classification may facilitate early identification of older adults at elevated risk (e.g., persistently low or declining participation) and help guide scalable responses, such as community-based activity programs and social prescribing, particularly in rural and resource-limited settings.

Although this study presents important findings, it has several limitations. Initially, since social participation and some covariates were self-reported, the findings may be subject to recall errors and response bias. Additionally, although social participation was quantified in detail, it was operationalized as a composite frequency-based score; therefore, we could not disentangle changes in specific activity types or domains (e.g., productive vs. recreational) or capture qualitative aspects (e.g., proactiveness or satisfaction). Consequently, we could not determine which activities declined most within the decreasing trajectory, or whether such reductions primarily reflected early cognitive decline (reverse causation) rather than other age-related constraints. Relatedly, although life events and role transitions (e.g., retirement, re-socialization after widowhood, grandparenting/caregiving, relocation, or changes in community opportunities) may contribute to increased participation in some older adults, we were unable to formally test these triggers because relevant measures were not consistently available across waves, and the timing of such events could not be aligned to establish temporal ordering. Future studies incorporating item-level measures and longitudinal designs (e.g., lagged analyses or excluding early cases) are warranted to clarify activity-specific patterns and better address directionality. Furthermore, the evaluation of cognitive impairment relied on scores derived from scales, which cannot entirely replace clinical diagnosis. In addition, comparisons between included and excluded eligible participants suggested potential selection bias; the analytic sample tended to be younger, more often male and urban, and had higher educational attainment, better baseline cognition and social participation, and a slightly lower prevalence of chronic disease, which may limit generalizability and could attenuate the observed associations. Lastly, as this is an observational study, despite using extensive adjustments for covariates, there remains a potential for residual confounding or reverse causation that cannot be dismissed.

## 5 Conclusion

This study identified five representative longitudinal patterns of social participation among older Chinese adults: “stable low”, “moderately declining”, “low baseline–increasing”, “stable intermediate” and “stable high”. Compared with those in the “stable low” group, individuals in the “stable high” and “stable intermediate” trajectories exhibited lower odds of cognitive impairment. Notably, participants in the “low baseline–increasing” trajectory also showed a lower odds of cognitive impairment, suggesting a potentially modifiable profile in which engagement can improve over time even among those initially disengaged. These findings underscore the importance of the dynamic evolution of social participation in shaping cognitive health and highlight its critical role in the prevention of cognitive impairment. Ongoing monitoring of changes in social engagement among older adults is therefore essential, with particular attention to those exhibiting declining participation. Targeted interventions—such as enhancing access to diverse social resources and providing emotional support—may help maintain or improve levels of engagement, ultimately contributing to better cognitive outcomes and the broader goal of healthy aging.

## Ethical standards

This research was conducted in accordance with the ethical principles outlined in the Declaration of Helsinki. Ethical approval for CHARLS was granted by the Biomedical Ethics Review Committee of Peking University (approval number: IRB00001052–11,015), and all participants gave written informed consent.

## Funding

This study was supported by the Key Research and Development Program funded by the Ministry of Science and Technology of the People’s Republic of China (No. 2023YFC3503701), the Joint Funds for the Innovation of Science and Technology, Fujian Province (No. 2024Y9531), the Scientific Research Fund for Top Youth Talents of Fujian University of Traditional Chinese Medicine (No. XQC2023005), and the Research Fund of Fujian University of Traditional Chinese Medicine (No. XJB2022007).

## Declaration of generative AI and AI-assisted technologies in the writing process

No form of artificial intelligence was utilized in the preparation of this manuscript.

## Data availability

The datasets analyzed during this study are publicly available from the CHARLS website: https://charls.pku.edu.cn.

## CRediT authorship contribution statement

**Kangle Wang:** Writing – original draft, Project administration, Formal analysis, Data curation, Conceptualization. **Ruihan Wan:** Writing – original draft, Methodology, Investigation, Data curation. **Jiale Peng:** Writing – original draft, Methodology, Investigation, Data curation. **Huanghao Zhou:** Writing – review & editing, Writing – original draft. **Kaifeng Xu:** Formal analysis. **Hao Liu:** Formal analysis. **Lidian Chen:** Writing – review & editing, Supervision, Conceptualization. **Zhizhen Liu:** Writing – review & editing, Supervision, Conceptualization.

## Declaration of competing interests

The authors declare that they have no known competing financial interests or personal relationships that could have appeared to influence the work reported in this paper.

## Appendix Supplementary materials

Image, application 1
