# Supplementary material for: Trajectories of social participation and risk of cognitive impairment in Chinese older adults: A six-year longitudinal study
Source: J Prev Alzheimers Dis. 2026 Jan 30;13(4):100499. doi: 10.1016/j.tjpad.2026.100499 (PMC12874414; doi:10.1016/j.tjpad.2026.100499)
Supplement: Supplementary file 1 [file mmc1.docx]

**Supplementary Materials**

**Title: Trajectories of social participation and risk of cognitive impairment in Chinese older adults: a six-year longitudinal study**

**Figure S1. Flowchart of participant selection.**

**Figure S2. The optimal trajectory curves of social participation over a six-year period(N=1326)**

**Table S1. Comparison of baseline characteristics between participants included in the analytic sample and eligible participants excluded from analysis (age ≥60 years).**

**Table S2. Fit indices of GBTM model on social participation trajectories**

**Table S3. Logistic regression results examining the association between social participation trajectories and cognitive impairment (ref: stable high group)**

**Table S4. Sensitivity analysis including baseline cognitive function (2013) in the fully adjusted model.**

**Table S5. Fit indices of GBTM model on BRI trajectories(N=1326)**

**Table S6. Logistic regression results examining the association between social participation trajectories and cognitive impairment(N=1326)**


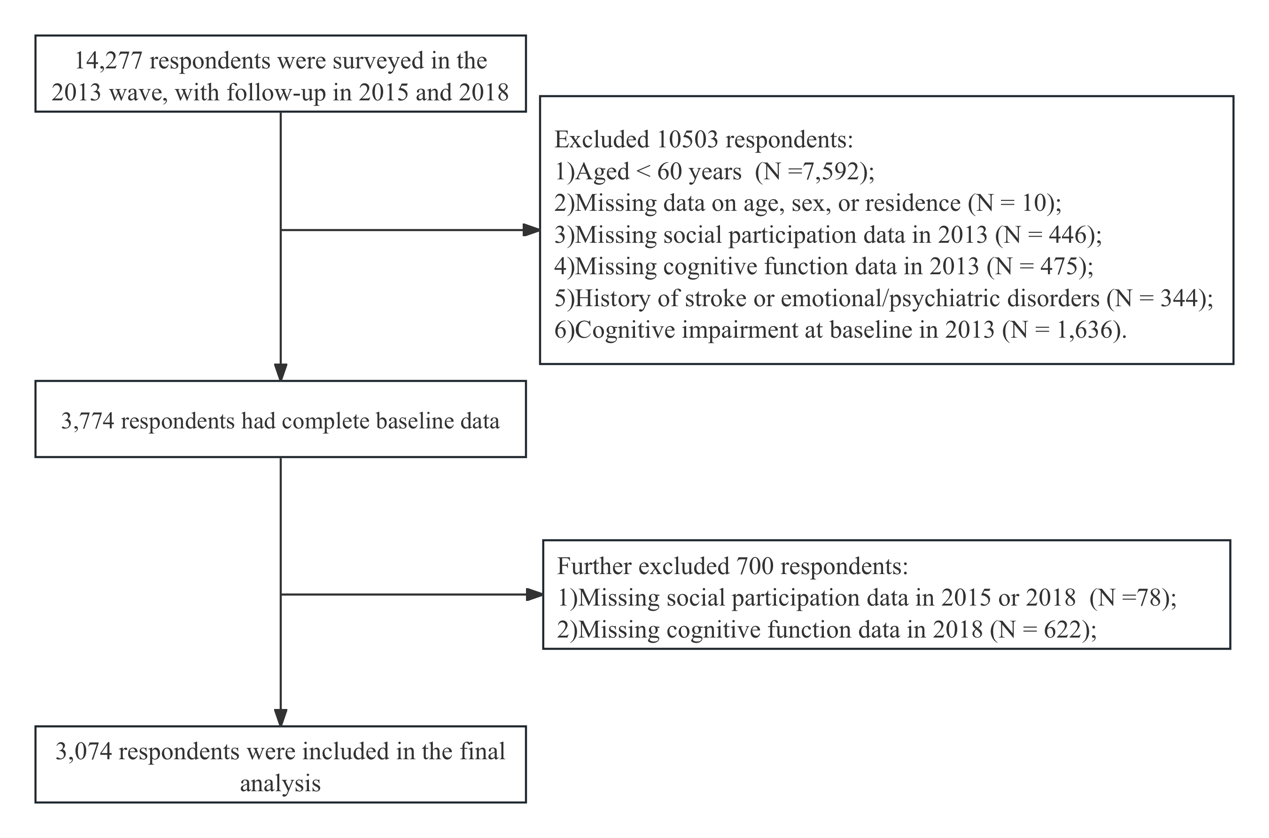


**Figure S1. Flowchart of participant selection.**


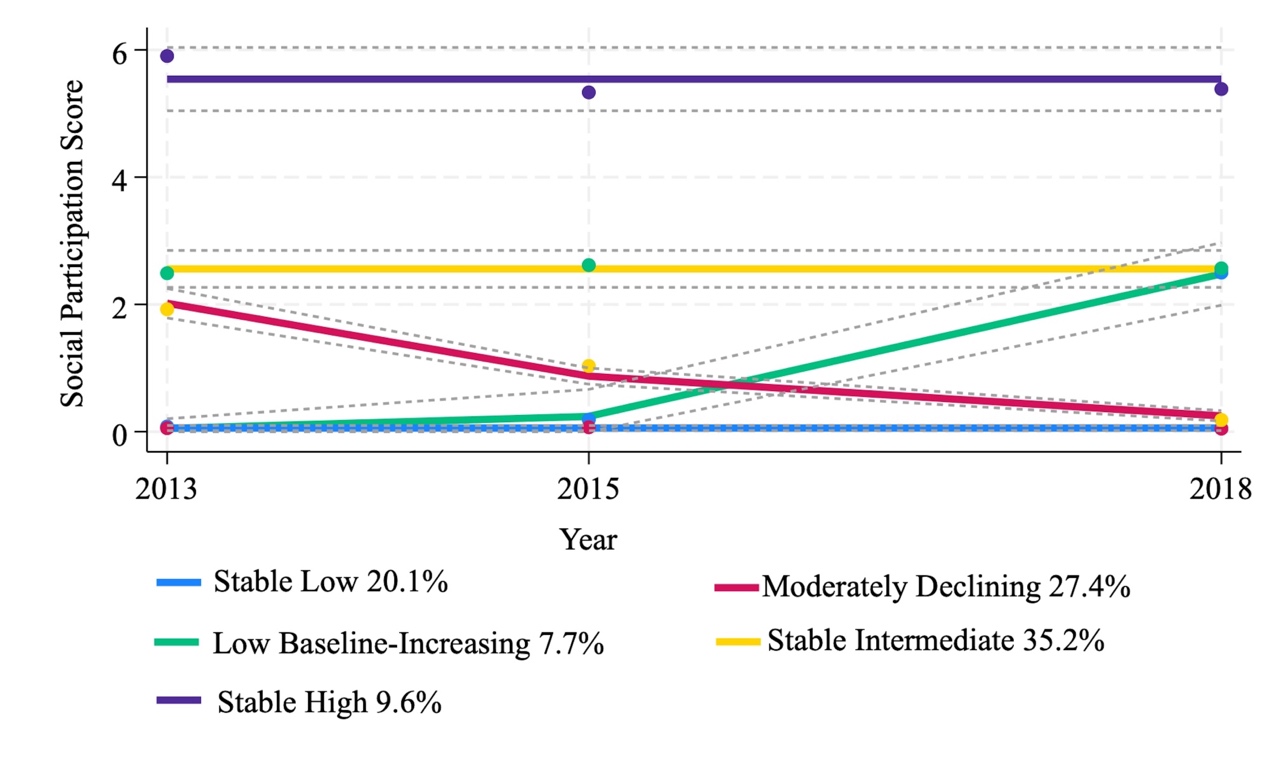


**Figure S2. Estimated social participation trajectories over a six-year follow-up period**

**(N=1326)**

Note: Solid lines depict model-estimated trajectories; dots represent observed group means at each wave; dashed lines indicate the 95% confidence intervals for the estimated values. Percentages in the legend represent model-estimated group proportions (TotProb).

**Table S1. Comparison of baseline characteristics between participants included in the analytic sample and eligible participants excluded from analysis (age ≥60 years).**

| Characteristics | Included (n = 3074) | Excluded (n = 3608) | P value |
| --- | --- | --- | --- |
| Age | 65.98 (5.05) | 69.06 (7.17) | <0.001 |
| Sex |  |  | <0.001 |
| Male | 1773 (57.7%) | 1453 (40.3%) |  |
| Female | 1301 (42.3%) | 2152 (59.6%) |  |
| Missing | 0 (0.0%) | 3 (0.1%) |  |
| Residence |  |  | <0.001 |
| Rural | 2735 (89.0%) | 3440 (95.3%) |  |
| City | 339 (11.0%) | 164 (4.5%) |  |
| Missing | 0 (0.0%) | 4 (0.1%) |  |
| BMI |  |  | <0.001 |
| Lean | 135 (4.4%) | 265 (7.3%) |  |
| Normal | 1552 (50.5%) | 1639 (45.4%) |  |
| Overweight | 682 (22.2%) | 601 (16.7%) |  |
| Obese | 117 (3.8%) | 130 (3.6%) |  |
| Missing | 588 (19.1%) | 973 (27.0%) |  |
| Education |  |  | <0.001 |
| Illiterate | 460 (15.0%) | 1887 (52.3%) |  |
| Primary school | 1770 (57.6%) | 1395 (38.7%) |  |
| Junior high school | 545 (17.7%) | 229 (6.3%) |  |
| Senior high school and above | 299 (9.7%) | 97 (2.7%) |  |
| Missing | 0 (0.0%) | 0 (0.0%) |  |
| Marital status |  |  | <0.001 |
| Married | 2672 (86.9%) | 2746 (76.1%) |  |
| Other | 399 (13.0%) | 858 (23.8%) |  |
| Missing | 3 (0.1%) | 4 (0.1%) |  |
| ADLs | 5.58 (0.90) | 5.33 (1.27) | <0.001 |
| Missing | 940 (28.8%) | 624 (18.3%) |  |
| IADLs | 5.56 (0.94) | 4.86 (1.52) | <0.001 |
| Missing | 0 (0.0%) | 38 (1.1%) |  |
| CESD_10 | 7.40 (5.43) | 8.96 (6.13) | <0.001 |
| Missing | 0 (0.0%) | 524 (15.3%) |  |
| Smoking |  |  | <0.001 |
| Yes | 460 (15.0%) | 249 (6.9%) |  |
| No | 1827 (59.4%) | 2165 (60.0%) |  |
| Missing | 787 (25.6%) | 1194 (33.1%) |  |
| Drink |  |  | <0.001 |
| Yes | 304 (9.9%) | 212 (5.9%) |  |
| No | 1875 (61.0%) | 2620 (72.6%) |  |
| Missing | 895 (29.1%) | 776 (21.5%) |  |
| Self-rated health status | 3.00 (3.00, 4.00) | 4.00 (3.00, 4.00) | <0.001 |
| Missing | 2 (0.1%) | 39 (1.1%) |  |
| Chronic disease |  |  | <0.001 |
| Yes | 698 (22.7%) | 952 (26.4%) |  |
| No | 2376 (77.3%) | 2630 (72.9%) |  |
| Missing | 0 (0.0%) | 26 (0.7%) |  |
| Cognitive2013 | 16.74 (3.60) | 9.15 (5.02) | <0.001 |
| Missing | 0 (0.0%) | 917 (26.8%) |  |
| Social participation 2013 | 2.09 (2.40) | 1.37 (1.83) | <0.001 |
| Missing | 0 (0.0%) | 447 (13.1%) |  |

Abbreviations: BMI = Body Mass Index; ADL = activities of daily living; IADL = instrumental activities of daily living; CESD-10 = 10-item Center for Epidemiologic Studies Depression Scale.

**Table S2. Fit indices of GBTM model on social participation trajectories**

| Group (Parameter) | BIC | aBIC | AIC | Entroy | Class (%) | AvePP |
| --- | --- | --- | --- | --- | --- | --- |
| 1Group (1) | -20998.9 | -20997.8 | -20991.77 | 1 | 100 | 1.00 |
| 2Group (1 1) | -17622.96 | -17620.21 | -17605.14 | 0.812 | 59.59/40.41 | 0.96/0.93 |
| 3Group (1 0 1) | -17050.4 | -17046.56 | -17025.45 | 0.771 | 32.63/48.41/18.96 | 0.91/0.89/0.90 |
| 4Group (0 1 1 1) | -16745.35 | -16739.86 | -16709.7 | 0.723 | 23.98/25.50/27.07/23.45 | 0.88/0.81/0.82/0.89 |
| 5Group (0 1 1 0 0) | -16546.97 | -16540.93 | -16507.76 | 0.740 | 19.95/27.95/11.98/32.71/7.42 | 0.86/0.82/0.79/0.85/0.88 |

Note: Parameter: 0= Intercept, 1= Linear, 2= Quadratic

Abbreviations: BIC, Bayesian Information Criterion; aBIC, adjusted Bayesian Information Criterion; AIC, Akaike Information Criterion; OCC indicates correct classification; AvePP , Fair posterior probability.

**Table S3. Logistic regression results examining the association between social participation trajectories and cognitive impairment (ref: stable high group)**

| Characteristic | Model 1 | | Model 2 | | Model 3 | | Model 4 | |
| --- | --- | --- | --- | --- | --- | --- | --- | --- |
|  | OR (95% CI) | P value | OR (95% CI) | P value | OR (95% CI) | P value | OR (95% CI) | P value |
| Social participation trajectories (ref: stable high) | |  |  |  |  |  |  |  |
| Stable low | 5.80(3.34,10.07) | <0.001 | 2.53(1.39,4.60) | 0.002 | 2.70(1.45,5.03) | 0.002 | 2.39(1.28,4.45) | 0.006 |
| Moderately declining | 4.61 (2.67,7.97) | <0.001 | 2.17(1.20,3.93) | 0.01 | 2.35(1.28,4.35) | 0.006 | 2.17(1.18,4.01) | 0.013 |
| Low baseline-increasing | 3.73(2.08,6.67) | <0.001 | 1.62(0.86,3.04) | 0.134 | 1.75(0.91,3.35) | 0.093 | 1.56(0.81,2.99) | 0.184 |
| Stable intermediate | 3.28(1.89,5.66) | <0.001 | 1.80(1.00,3.25) | 0.051 | 1.94(1.06,3.59) | 0.033 | 1.82(0.98,3.36) | 0.054 |
| Age |  |  | 1.06(1.04,1.08) | <0.001 | 1.06(1.04,1.08) | <0.001 | 1.05(1.03,1.07) | <0.001 |
| Female (ref: male) |  |  | 1.09(0.90,1.33) | 0.389 | 1.30(1.04,1.64) | 0.024 | 1.24(0.98,1.56) | 0.073 |
| City (ref: rural) |  |  | 0.40(0.26,0.64) | <0.001 | 0.40(0.25,0.63) | <0.001 | 0.42(0.27,0.67) | <0.001 |
| Married (ref: other) |  |  | 0.74(0.57,0.96) | 0.026 | 0.76(0.58,0.99) | 0.040 | 0.80(0.61,1.04) | 0.099 |
| Education (ref: illiterate) |  |  |  |  |  |  |  |  |
| Primary school |  |  | 0.17(0.14,0.22) | <0.001 | 0.17(0.14,0.22) | <0.001 | 0.18(0.14,0.23) | <0.001 |
| Junior high school |  |  | 0.05(0.04,0.08) | <0.001 | 0.05(0.03,0.07) | <0.001 | 0.05(0.04,0.08) | <0.001 |
| Senior high school and above |  |  | 0.03(0.01,0.05) | <0.001 | 0.02(0.01,0.05) | <0.001 | 0.03(0.01,0.05) | <0.001 |
| Smoke (ref: no) |  |  |  |  | 1.37(1.07,1.75) | 0.011 | 1.29(1.01,1.65) | 0.043 |
| Drink (ref: no) |  |  |  |  | 0.83(0.67,1.03) | 0.094 | 0.83(0.67,1.03) | 0.097 |
| BMI (ref: lean) |  |  |  |  |  |  |  |  |
| Normal |  |  |  |  |  |  | 0.58(0.39,0.86) | 0.007 |
| Overweight |  |  |  |  |  |  | 0.54(0.35,0.83) | <0.001 |
| Obese |  |  |  |  |  |  | 0.45(0.25,0.83) | 0.01 |
| ADL |  |  |  |  |  |  | 1.05(0.95,1.17) | 0.312 |
| IADL |  |  |  |  |  |  | 0.86(0.77,0.96) | 0.005 |
| CESD-10 |  |  |  |  |  |  | 1.03(1.01,1.05) | 0.005 |
| Self-rated health status |  |  |  |  |  |  | 1.00(0.90,1.11) | 0.972 |
| With chronic disease (ref: no) |  |  |  |  |  |  | 0.92(0.73,1.17) | 0.501 |

Note: logistic models estimated the association between different social participation trajectories and cognitive impairment risk, using the stable high group as reference. Model 1 included no covariates; Model 2 adjusted for sociodemographic factors; Model 3 incorporated health behaviors; Model 4 further included physical health and psychological well-being indicators. OR = odds ratio; CI = confidence interval.

**Table S4. Sensitivity analysis including baseline cognitive function (2013) in the fully adjusted model.**

| Social participation trajectory | OR (95% CI) | P value |
| --- | --- | --- |
| Stable low(ref) |  |  |
| Moderately declining | 1.01 (0.77, 1.31) | 0.972 |
| Low baseline-increasing | 0.68 (0.49, 0.96) | 0.030 |
| Stable intermediate | 0.85 (0.66, 1.12) | 0.247 |
| Stable high | 0.55 (0.29, 1.04) | 0.068 |

Note: Adjusted for the same covariates as the primary fully adjusted model (sociodemographic, health-related, and behavioral factors) with additional adjustment for baseline cognitive score (2013).

**Table S5. Fit indices of GBTM model on BRI trajectories (N=1326)**

| Group (Parameter) | BIC | aBIC | AIC | Entroy | Class (%) | AvePP |
| --- | --- | --- | --- | --- | --- | --- |
| 1Group (1) | -8696.8 | -8695.7 | -8690.51 | 1 | 100 | 1.00 |
| 2Group (1 0) | -7389.54 | -7387.34 | -7376.96 | \| \| 0.795 \| \| --- \| \| \| --- \| --- \| | 57.27/42.73 | 0.94/0.94 |
| 3Group (1 0 0) | -7168.89 | -7165.59 | -7150.02 | 0.763 | 34.60/48.73/16.67 | 0.90/0.89/0.89 |
| 4Group (0 1 1 1) | -7053.58 | -7048.09 | -7022.14 | 0.71 | 23.93/25.81/28.44/21.82 | 0.86/0.80/0.82/0.88 |
| 5Group (1 0 0 1 0) | -6985.3 | -6979.25 | -6950.71 | 0.741 | 7.66/20.11/35.22/27.39/9.62 | 0.77/0.91/0.84/0.78/0.88 |

Note: Parameter: 0= Intercept, 1= Linear, 2= Quadratic

Abbreviations: BIC, Bayesian Information Criterion; aBIC, adjusted Bayesian Information Criterion; AIC, Akaike Information Criterion; OCC indicates correct classification; AvePP , Fair posterior probability.

**Table S6. Logistic regression results examining the association between social participation trajectories and cognitive impairment (N=1326)**

| Characteristic | Model 1 | | Model 2 | | Model 3 | | Model 4 | |
| --- | --- | --- | --- | --- | --- | --- | --- | --- |
|  | OR (95% CI) | P value | OR (95% CI) | P value | OR (95% CI) | P value | OR (95% CI) | P value |
| Social participation trajectories (ref: stable low group) | | | |  |  |  |  |  |
| Low baseline-increasing | 0.69 (0.42, 1.12) | 0.137 | 0.71 (0.41, 1.19) | 0.199 | 0.72 (0.42, 1.22) | 0.223 | 0.72 (0.41, 1.22) | 0.223 |
| Stable intermediate | 0.58 (0.42, 0.80) | <0.001 | 0.64 (0.44, 0.91) | 0.015 | 0.63 (0.44, 0.91) | 0.013 | 0.67 (0.46, 0.97) | 0.033 |
| Moderately declining | 0.68 (0.49, 0.95) | 0.025 | 0.70 (0.48, 1.01) | 0.059 | 0.69 (0.48, 1.00) | 0.053 | 0.73 (0.50, 1.06) | 0.095 |
| Stable high | 0.19 (0.09, 0.35) | <0.001 | 0.39 (0.19, 0.77) | 0.009 | 0.39 (0.18, 0.76) | 0.008 | 0.43 (0.20, 0.86) | 0.022 |
| Age |  |  | 1.05 (1.02, 1.08) | <0.001 | 1.05 (1.02, 1.08) | <0.001 | 1.05 (1.02, 1.08) | <0.001 |
| Male (ref: female) |  |  | 1.08 (0.81, 1.44) | 0.592 | 0.92 (0.65, 1.3) | 0.633 | 0.95 (0.67, 1.35) | 0.789 |
| Education (ref: illiterate) | |  |  |  |  |  |  |  |
| Primary school |  |  | 0.17 (0.13, 0.24) | <0.001 | 0.17 (0.13, 0.24) | <0.001 | 0.18 (0.13, 0.24) | <0.001 |
| Junior high school |  |  | 0.05 (0.02, 0.09) | <0.001 | 0.05 (0.03, 0.09) | <0.001 | 0.05 (0.03, 0.10) | <0.001 |
| Senior high school and above | |  | 0.04 (0.01, 0.08) | <0.001 | 0.04 (0.01, 0.08) | <0.001 | 0.04 (0.02, 0.10) | <0.001 |
| Married (ref: other) |  |  | 0.77 (0.53, 1.13) | 0.173 | 0.79 (0.54, 1.15) | 0.216 | 0.82 (0.56, 1.21) | 0.311 |
| City (ref: rural) |  |  | 0.50 (0.26, 0.93) | 0.036 | 0.50 (0.25, 0.93) | 0.037 | 0.52 (0.26, 0.96) | 0.045 |
| Smoke (ref: no) |  |  |  |  | 1.34 (0.90, 2.00) | 0.147 | 1.20 (0.80, 1.81) | 0.379 |
| Drink (ref: no) |  |  |  |  | 1.14 (0.83, 1.57) | 0.407 | 1.14 (0.82, 1.56) | 0.434 |
| Self-rated health status |  |  |  |  |  |  | 1.02 (0.88, 1.19) | 0.808 |
| ADLs |  |  |  |  |  |  | 1.15 (0.96, 1.38) | 0.136 |
| IADLs |  |  |  |  |  |  | 0.85 (0.73, 0.99) | 0.033 |
| BMI (ref: lean) |  |  |  |  |  |  |  |  |
| Normal |  |  |  |  |  |  | 0.57 (0.33, 1.00) | 0.050 |
| Overweight |  |  |  |  |  |  | 0.49 (0.27, 0.89) | 0.020 |
| Obese |  |  |  |  |  |  | 0.49 (0.22, 1.08) | 0.081 |
| CESD_10 |  |  |  |  |  |  | 1.02 (1.00, 1.05) | 0.070 |
| With chronic disease (ref: no) |  |  |  |  |  |  | 0.97 (0.68, 1.40) | 0.862 |

Note: Logistic regression models were used to examine the association between social participation trajectories and the risk of cognitive impairment, with the stable low group as the reference category. Model 1 included no covariates; Model 2 adjusted for sociodemographic factors; Model 3 incorporated health behaviors; Model 4 further included physical health and psychological well-being indicators. OR = odds ratio; CI = confidence interval.
